# Device Design Implications on *R*_ON_×*Q*_OSS_ FOM in p-GaN Power HEMTs: A Simulation Study

**DOI:** 10.3390/mi17101182

**Published:** 2026-10-13

**Authors:** Nicolò Zagni, Giovanni Verzellesi, Alessandro Chini

**Affiliations:** 1 Department of Engineering “Enzo Ferrari” (DIEF), University of Modena and Reggio Emilia, Via P. Vivarelli 10, 41125 Modena, Italy; 2 Department of Science and Methods for Engineering and EN&TECH, University of Modena and Reggio Emilia, Via G. Amendola 2, 42122 Reggio Emilia, Italy

**Keywords:** p-GaN HEMT, dynamic *R*_ON_, dynamic *Q*_OSS_, *R*_ON_×*Q*_OSS_, C-doping, hole redistribution model, TCAD simulations, resonant converter, efficiency

## Abstract

In this paper, we analyze by means of numerical simulations the device design implications on the *R*_ON_×*Q*_OSS_ Figure of Merit (FOM) in p-GaN power HEMTs to be employed in soft-switching converters. During switching, p-GaN power HEMTs experience dynamic *R*_ON_ increase but also dynamic *Q*_OSS_ reduction, effectively establishing a trade-off between the two device parameters. This trade-off stems from the physical mechanism of hole redistribution within the C-doped buffer. Device design choices such as field plate length, channel and buffer thickness as well as C-doping concentration influence both parameters; however, the relative variation of the two parameters is not necessarily symmetric. Simulations are therefore carried out to identify the best device design choices depending on the target trade-off between *R*_ON_ and *Q*_OSS_.

## 1. Introduction

**Figure 1:**
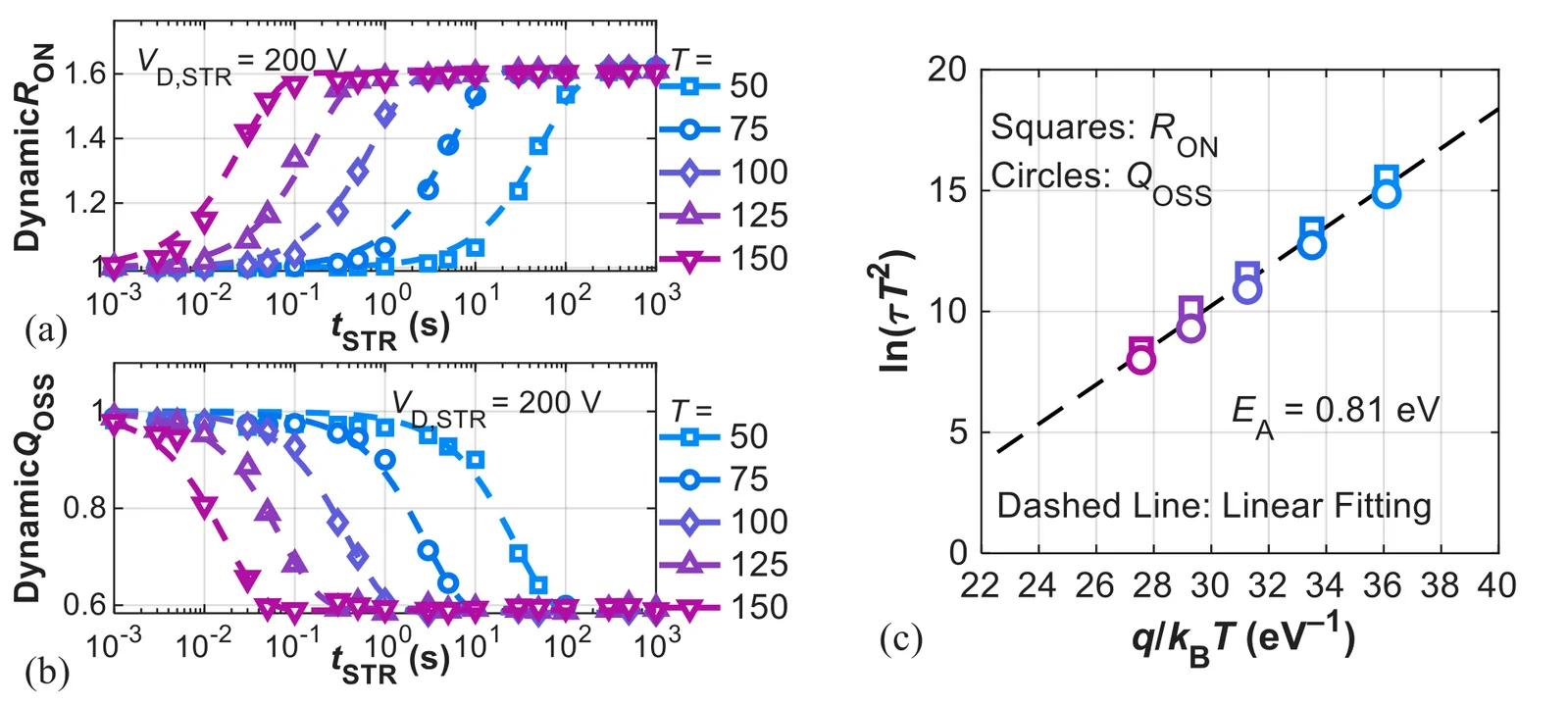
Dynamic (**a**) *R*_ON_ and (**b**) *Q*_OSS_ transients. Simulations were carried out at *V*_D,STR_ = 200 V at different temperatures, *T* = 50–150 °C (Δ*T* = 25 °C). Dashed lines are the stretched-exponential fitting curves of the data. (**c**) Arrhenius plot extracted from the dynamic *R*_ON_ and *Q*_OSS_ transients. The dashed line is the linear fitting of the data, from which an *E*_A_ = 0.81 eV was extracted in both cases, correlating with the C_N_ trap level (0.9 eV).

Gallium Nitride (GaN) has established its presence among wide-band-gap semiconductors for applications mainly in the power electronics and RF domains [1,2,3]. The advantages of GaN-based devices over other material systems stem from its excellent intrinsic material properties but also from the High Electron Mobility Transistor (HEMT) structure, which effectively enables high-voltage and high-frequency operation. From a device point of view, GaN-based HEMTs offer both low conduction losses (i.e., low *R*_ON_) and low switching losses (i.e., low *Q*_G_), with E-mode p-GaN HEMTs having the best *R*_ON_×*Q*_G_ figure of merit amongst commercial devices [4]. Notwithstanding these merits, GaN-based devices suffer from significant dispersion effects related to trapping that affect their stability and reliability during operation [5]. One of the primary sources for dispersion is the traps in the buffer introduced by C-doping in the buffer [1], which are energetically located at about 0.9 eV above the GaN valence band [6]. The cause of dispersion is essentially the exchange of holes with the valence band, a process which exhibits a long time constant, typically in the order of tens of seconds at *T* = 30 °C [1,6,7]. The emission and redistribution of holes within the buffer modulate the number of ionized traps in the buffer itself, in turn modulating the two-dimensional electron gas (2DEG) concentration and therefore affecting the ON-state resistance (*R*_ON_) [6]. Recently, we showed how the hole redistribution in the buffer also modulates the output charge (*Q*_OSS_ = *Q*_GD_ + *Q*_DS_) as this quantity also depends on 2DEG concentration (i.e., the non-linear, non-constant output capacitance *C*_OSS_ = *C*_GD_ + *C*_DS_ varies if 2DEG varies) [8,9]. The correlation between dynamic *R*_ON_ and *Q*_OSS_ was first attributed to C-related traps in the buffer by the experimental study of Cioni et al. [8]. In the study, a simultaneous on-the-fly characterization of the two parameters was performed under switching conditions at 200 V. It was shown how dynamic *R*_ON_ increased with time while dynamic *Q*_OSS_ decreased, and more specifically it was observed how the variation of the two parameters followed an exponential evolution over time with approximately equal time constants in a wide temperature range from 25 to 150 °C. This behavior was reproduced with numerical simulations in our previous work, where it was shown how both dynamic *R*_ON_ increase and dynamic *Q*_OSS_ decrease could be explained with the hole redistribution model [9]. Figure 1 summarizes these findings by showing how the activation energy (*E*_A_) of dynamic *R*_ON_ (*Q*_OSS_) increase (decrease) is 0.81 eV, which correlates with the C-related trap energy level (0.9 eV). Dynamic *R*_ON_ and *Q*_OSS_ are defined as relative values with respect to the value prior to stress, i.e., dynamic *R*_ON_ = *R*_ON_(*t*)/*R*_ON_(0), dynamic *Q*_OSS_ = *Q*_OSS_(*t*)/*Q*_OSS_(0).

In this work, we build upon the findings of [9] to show the effects of different design choices on the relative trade-off between *R*_ON_ and *Q*_OSS_. Therefore, while the conference paper in ref. [9] focuses on clarifying the physical mechanism behind dynamic *R*_ON_ and *Q*_OSS_ correlation, namely the hole redistribution due to C-related traps, here the focus is on the design implications on the trade-off mentioned above, and on the possible strategies to minimize the FOM *R*_ON_×*Q*_OSS_, as discussed in the following.

We employ numerical simulations to show the sensitivity of both *R*_ON_ and *Q*_OSS_ on channel, barrier, and buffer thickness, carbon concentration, field plate(s) length, and passivation(s) thickness. Here, we focus on *Q*_OSS_ = *Q*_GD_ + *Q*_DS_ rather than *Q*_G_ (or *Q*_GD_ only) because in soft-switching converters *Q*_OSS_ is more detrimental to losses than *Q*_G_, especially at high voltage ratings [10], and switching losses are almost absent (conversely, in hard-switching conditions, *Q*_GD_ is the limiting factor). More specifically, in soft-switching and resonant converters, switching losses are almost completely removed by including a dead-time interval during the period to ensure zero-voltage switching (ZVS) conditions. However, longer dead-time duration leads to higher conduction losses as the RMS current needed to transfer power to the output has to increase if the duty cycle reduces [10]. Dead-time duration is limited by *Q*_OSS_ (i.e., it corresponds to the time taken to discharge the non-linear output capacitance *C*_OSS_), since the transistor drain-to-source voltage needs to be brought to zero volts in order to have ZVS. Therefore, design of GaN HEMTs for power converters working in soft-switching conditions should aim to minimize *R*_ON_×*Q*_OSS_ [10,11,12].

## 2. Simulation Setup

**Figure 2:**
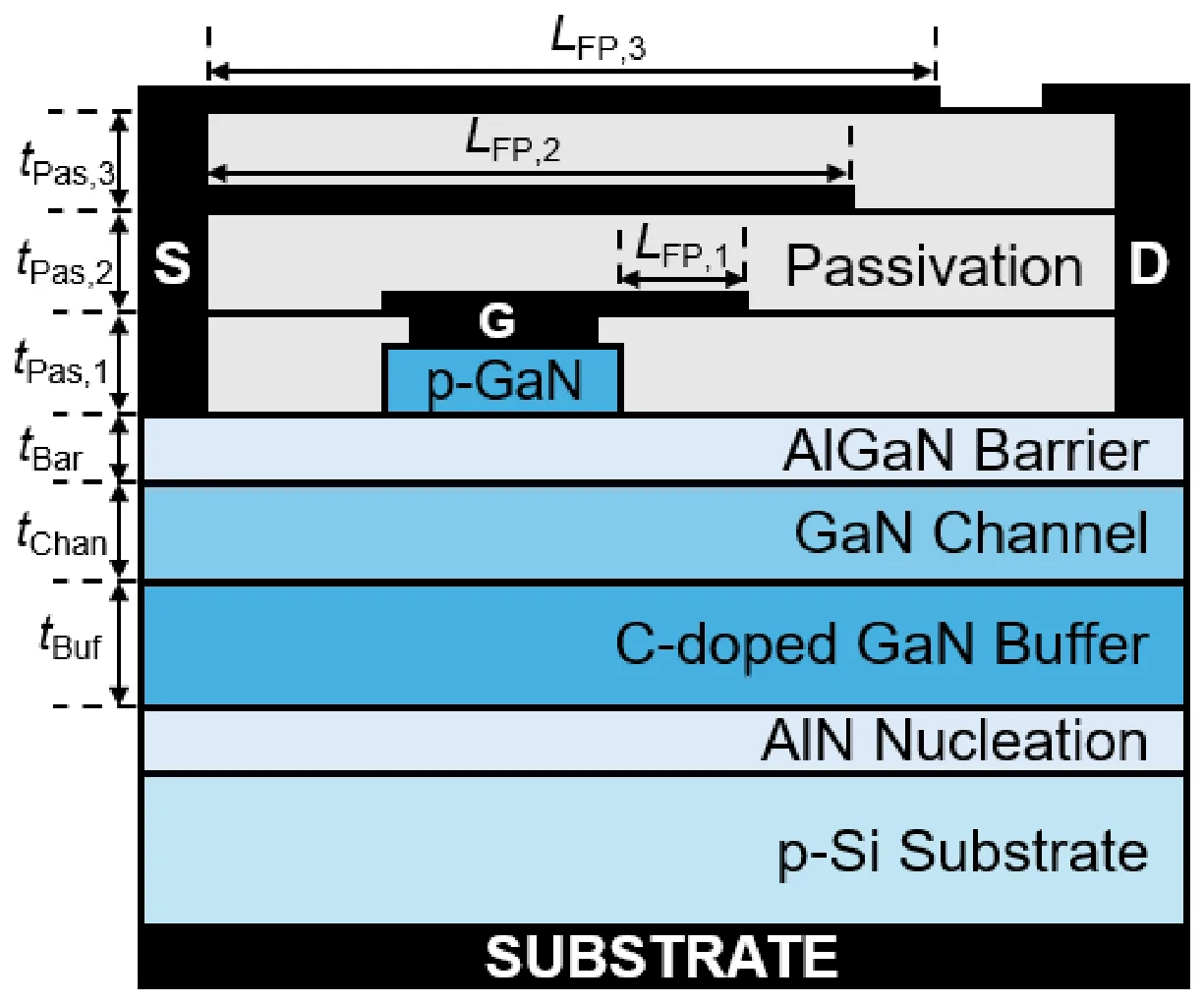
Cross-section of the simulated 2D device. The geometrical parameters subject to variation in the analysis are indicated.

In this section we describe the numerical simulation setup in terms of device structure, models, and main simulation parameters. The structure is shown in Figure 2, which resembles a typical p-GaN HEMT for power applications. Note in particular the three separate field plates used to shield the drain edge of the gate terminal from large electric field peaks [13]. Note that field plate 1 is connected to the gate terminal, whereas field plates 2 and 3 are connected to the source terminal. The drain overhang is used to reduce the electric field peak at the drain terminal occurring at very large reverse voltages. Consistent with the structure in [7], which served as a template for the simulated device, the substrate was realized out of conductive p-type silicon, which is short-circuited to the source and thus grounded to the reference potential (0 V).

The numerical model is described as follows. The 2D numerical device simulations were carried out with the SDevice simulator (Synopsys Inc., Sunnyvale, CA, USA). Drift-diffusion was the model used to account for charge transport. Mobility was set to 1800 cm^2^/Vs in the 2DEG, exponentially decaying with temperature. Piezoelectric polarization charge was included at the AlGaN/GaN interface by using the default strain model of the simulator, leading to a 2DEG concentration of ≈7 × 10^12^ cm^−2^. Note that at the passivation/barrier interface, the polarization model was deactivated (i.e., we assumed that the negative polarization charge at this interface is completely compensated by an equal positive surface charge). Incomplete ionization of Mg-related acceptors in the p-GaN gate region was included in the model (the Mg ionization energy was set to 0.16 eV above the GaN valence band edge). Gate leakage was accounted for through hole tunneling across the Schottky junction formed by the gate metal contact and the p-GaN region. Source and drain contacts were modeled as Schottky junctions, with a similar approach to that discussed in [14]. Impact ionization was modeled with Chynoweth’s law. To model the vertical leakage conduction through the substrate and nucleation layers, we employed a similar strategy to that discussed in [15]. Dynamic effects related to C-doping traps were accounted for by adding one Shockley–Read–Hall (SRH) trap-balance equation for each distinct trap. Carbon doping in the GaN buffer was modeled by considering two different trap levels: a dominant deep acceptor trap at *E*_V_ + 0.9 eV and a shallow donor trap at *E*_C_ − 0.11 eV. The nominal trap concentrations were 4.3 × 10^17^ and 4 × 10^17^ cm^−3^ for C-related acceptors and donors, respectively. This corresponds to an effective acceptor density (*N*_C,Eff_) of 3 × 10^16^ cm^−3^. This particular choice is in line with what was done in ref. [7] to have a quantitative agreement with experimental results regarding dynamic *R*_ON_. Table 1 collects the relevant parameter values used for the reference device.

**Table 1:** Relevant parameter values used in the simulations for the reference device.

Parameter	Symbol	Value
Channel thickness	*t*_Chan_	0.3 µm
Barrier thickness	*t*_Bar_	12 nm
Buffer thickness	*t*_Buf_	4.7 µm
Field plate 1 length	*L*_FP,1_	3 µm
Field plate 2 length	*L*_FP,2_	10 µm
Field plate 3 length	*L*_FP,3_	15 µm
Passivation 1 thickness	*t*_Pas,1_	0.25 µm
Passivation 2 thickness	*t*_Pas,2_	0.25 µm
Passivation 3 thickness	*t*_Pas,3_	1 µm
Effective C-related trap concentration	*N*_C,Eff_	3 × 10^16^ cm^−3^
2DEG mobility	*µ*_n,2DEG_	1800 cm^2^/Vs
2DEG concentration	*n*_2DEG_	7 × 10^12^ cm^−2^

## 3. Numerical Simulation Results

The results described in the following are obtained from numerical simulations that mimicked the experimental conditions used in [8] to characterize dynamic *R*_ON_ and *Q*_OSS_. The simulated experiment consisted of the OFF-state stress condition applied for 1000 s, after which *R*_ON_ and *Q*_OSS_ were evaluated. *R*_ON_ was probed by switching the device ON (i.e., *V*_GS_ = 6 V, *V*_DS_ = 1 V) for 5 μs. *Q*_OSS_ was instead probed by switching *V*_DS_ from the stress value to 0 V within a 25 μs time window, during which *Q*_OSS_ was obtained by integrating *I*_D_ over time. Specifically,

$$Q_{OSS}=\int_{t(V_{DS}=V_{DS,STR})}^{t(V_{DS}=0)}I_{D}\left(t\right)dt$$

The *Q*_OSS_ thus defined represents the total output charge. Because the *Q*_OSS_ is taken while the output capacitance discharges, the sign of the current *I*_D_ is negative (and thus also *Q*_OSS_); therefore, the absolute value is taken.

**Figure 3:**
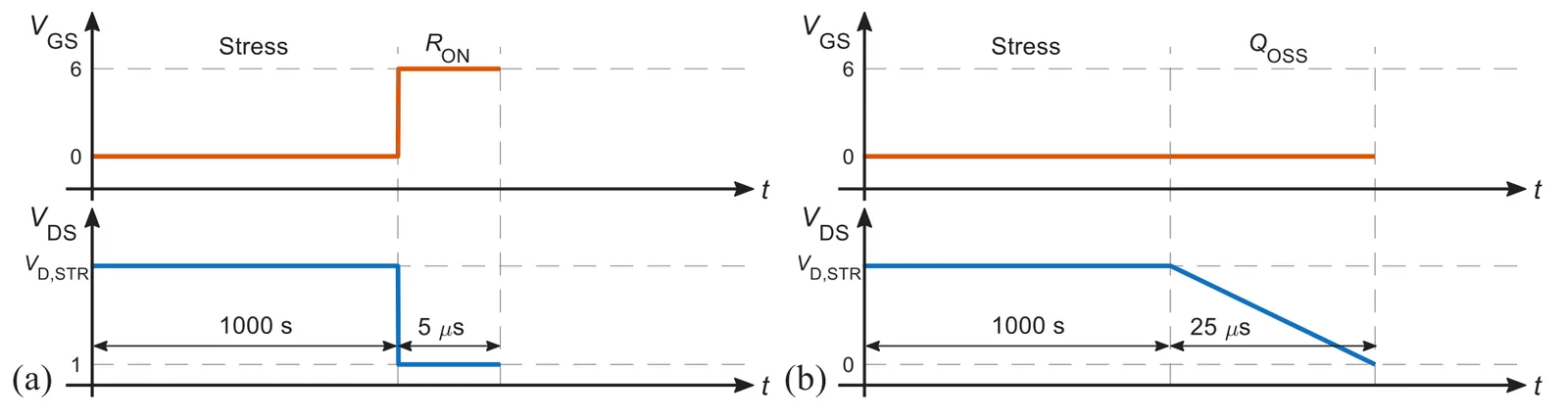
Sketch of *V*_GS_ and *V*_DS_ waveforms applied during simulations to extract (**a**) *R*_ON_ and (**b**) *Q*_OSS_ after stress. After application of OFF-state stress conditions for 1000 s, (**a**) assessment of *R*_ON_ is carried out in the ON-state for 5 µs, and (**b**) assessment of *Q*_OSS_ is carried out during switching of *V*_DS_ to 0 V for 25 µs. The time diagrams are not to scale and are for illustration only.

Figure 3 shows a sketch of the waveforms employed in the simulations to assess *R*_ON_ and *Q*_OSS_ during the simulated experiments. The on-pulse duration of 5 µs to monitor *R*_ON_ and the 25 µs-long ramp to bring *V*_DS_ from *V*_DS,STR_ to 0 V were set in agreement with the experimental setup defined in [8]. While the on-pulse duration is exactly the same as that in [8], the ramp duration agrees with the typical ramp duration in the experiments, which, however, is not defined *a priori*, because the drain potential is set by an external generator through a series resistance which is used to monitor the current during the switch-OFF. In the simulations, however, the drain potential is set directly; therefore, the ramp duration is given as an input. Nonetheless, neither the on-pulse nor the ramp time influences the *R*_ON_ and *Q*_OSS_ probing as long as their duration is well below the time constant of the hole emission process which defines the trap charge state (at *T* = 30 °C, the time constant is typically in the order of tens of seconds; therefore, this requirement is satisfied by the waveform durations set in the simulations). Stress duration of 1000 s was set in agreement with experimental conditions used in [8].

*R*_ON_ and *Q*_OSS_ were also evaluated after applying stress only for 10 µs in order to compare the *R*_ON_×*Q*_OSS_ with commercial devices. We refer to the values extracted in this way as ‘prior to stress’, since C-related traps are well known to cause negligible dispersion effects at such short time scales at *T* = 30 °C, see for instance [6,7]. Therefore, *R*_ON_ and *Q*_OSS_ extracted in this way are only affected by the electrostatics of the device and not by dynamic effects associated with traps.

Table 2 shows the comparison with state-of-the-art GaN HEMTs rated at 650 V. In the simulations, *R*_ON_ is extracted at *V*_GS_ = 6 V, *V*_DS_ = 1 V. *Q*_OSS_ is extracted instead at *V*_GS_ = 0 V, *V*_DS_ = 400 V. The *R*_ON_×*Q*_OSS_ extracted from the simulations is in the range of reported commercial technologies, providing a useful benchmark for the simulated device. Notice that the simulated device has a width of 1 mm; therefore, to make the simulated *R*_ON_ value comparable with those of commercial devices, a scaling factor of 200 (commercial devices have widths as large as hundreds or thousands of mm) was used to divide *R*_ON_; *Q*_OSS_ was then obtained by multiplying by the same factor of 200. Nevertheless, *R*_ON_×*Q*_OSS_ remains unaffected by scaling.

The nominal breakdown voltage (*V*_BR_) of the simulated device is 877 V. However, generally commercial devices are de-rated with respect to their actual *V*_BR_ for reliability concerns; therefore, the simulated device should be considered as a 26% de-rated 650 V device. Even so, the comparison with commercial technology is intended as a technology benchmark for *R*_ON_×*Q*_OSS_ rather than a direct device-to-device comparison.

**Table 2:** Comparison of *R*_ON_×*Q*_OSS_ of different commercial devices (values obtained from datasheets of vendors) rated for 650 V. The value extracted from the simulated device is in line with the state of the art.

Device	*R*_ON_ (mΩ)	*Q*_OSS_ (nC)	*R*_ON_×*Q*_OSS_ (nΩ C)
GAN041-650WSB (Nexperia)	35	150	5.25
SGT070R70HTO (ST) *	53	94.7	5.02
GS66516T (GaN System, now Infineon)	25	134	3.35
NTBL050N65GN1 (onsemi)	39	107	4.17
INN650D080BS (Innoscience)	80	60	4.8
TP65H050G4WS (Renesas)	50	120	6.0
IGLT65R055D2 (Infineon)	55	35	1.9
Simulated Device **	63.3 (12.66 Ωmm)	40.4 (0.202 nC/mm)	2.55

* Rated at 700 V. ** Width of simulated device is 1 mm; therefore, to make simulated *R*_ON_ and *Q*_OSS_ values comparable with those of commercial devices, a scaling factor of 200 was used (*R*_ON_×*Q*_OSS_ remains unaffected).

### 3.1. Results After 1000-s Stress

**Figure 4:**
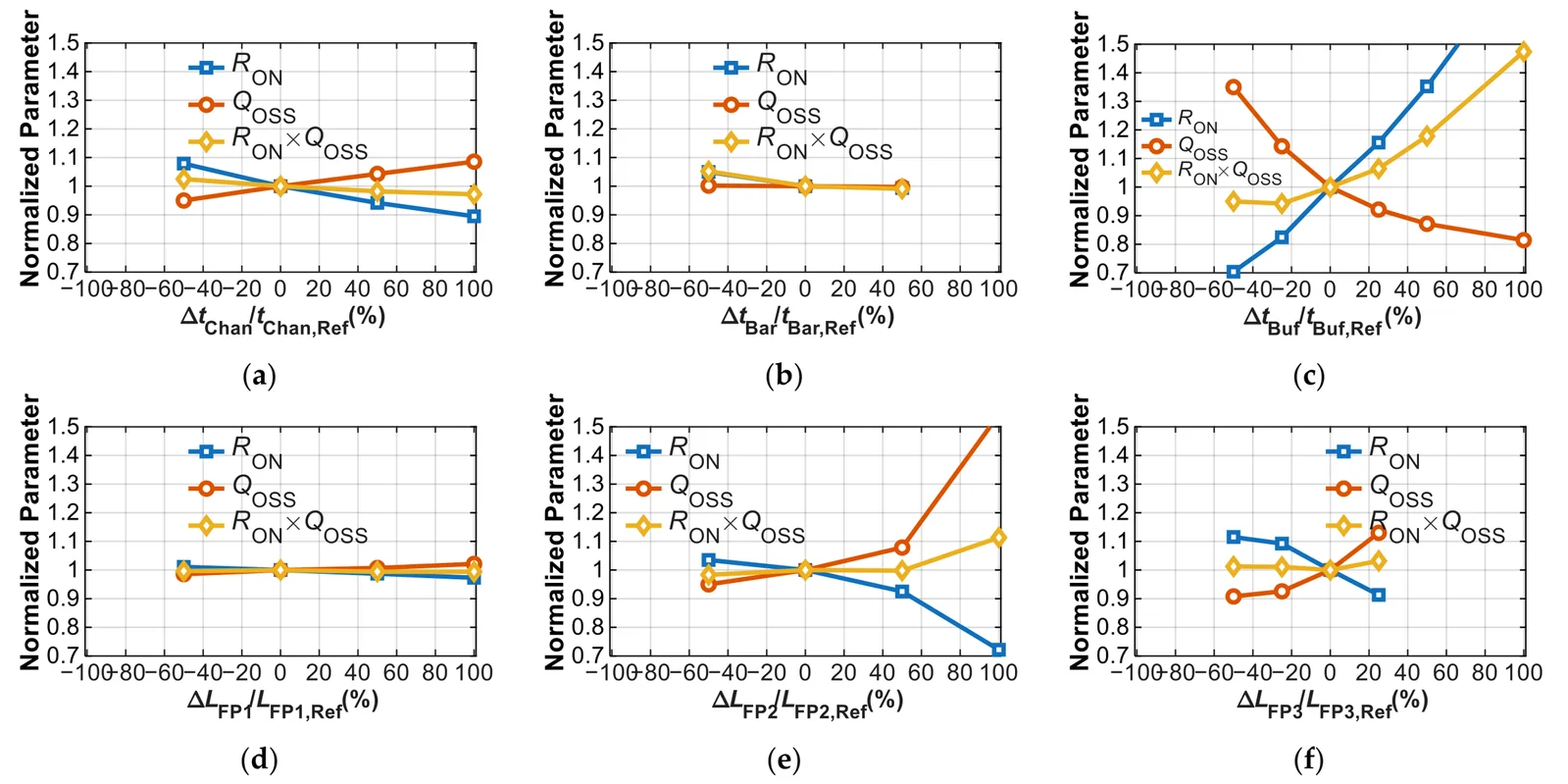

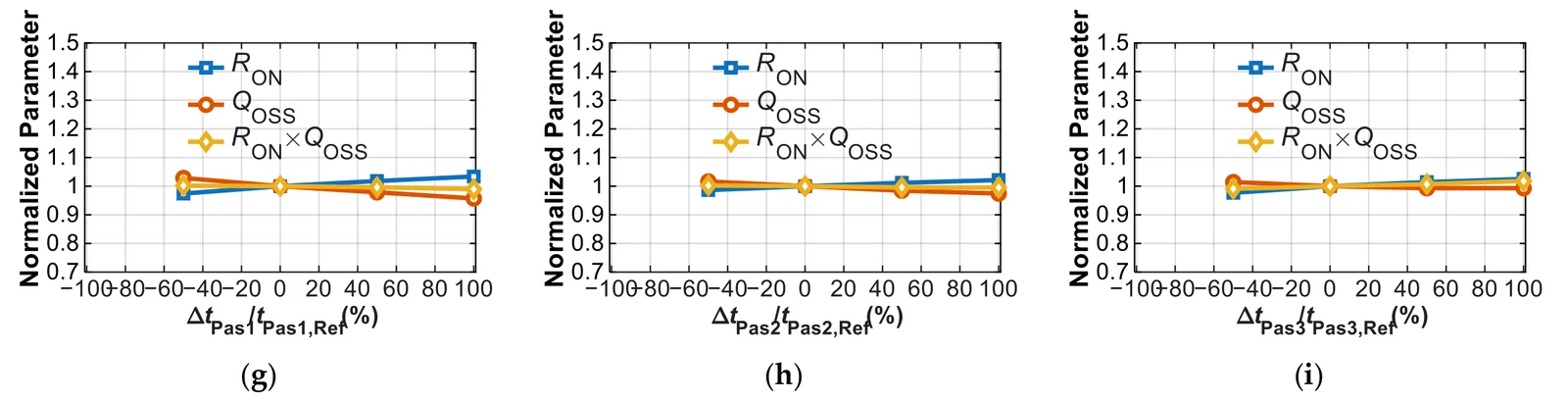
Sensitivity of *R*_ON_, *Q*_OSS_, and *R*_ON_×*Q*_OSS_ on variation of geometric parameters: (**a**) channel thickness, *t*_Chan_; (**b**) barrier thickness, *t*_Bar_; (**c**) buffer thickness, *t*_Buf_; (**d**) field plate 1 length (*L*_FP,1_); (**e**) field plate 2 length (*L*_FP,2_); (**f**) field plate 3 length (*L*_FP,3_); (**g**) passivation 1 thickness, *t*_Pas,1_; (**h**) passivation 2 thickness, *t*_Pas,2_; (**i**) passivation 3 thickness, *t*_Pas,3_. Parameters were evaluated at the end of the stress phase, with *V*_D,STR_ = 400 V applied for 1000 s at *T* = 30 °C.

Results of the sensitivity analysis are presented in Figure 4. Each panel shows the dependence of *R*_ON_, *Q*_OSS_, and *R*_ON_×*Q*_OSS_ on a single geometric parameter (all the others were kept fixed at their nominal value, reported in Table 1). The values are normalized to that of the reference device for a more immediate comparison. The values reported in Figure 4 are those taken at the end of the stress experiment illustrated in Figure 3 (the values prior to the stress experiment are reported in Section 3.2).

Figure 4a shows the effect of channel thickness (*t*_Chan_) variation: *t*_Chan_ increase moves the C-related traps away from the 2DEG, thereby reducing their influence on the channel electrons, hence *R*_ON_ decreases and at the same time *Q*_OSS_ increases. This leads to an almost constant *R*_ON_×*Q*_OSS_ (it decreases by about 3%). By the same token, a thinner *t*_Chan_ increases *R*_ON_ and decreases *Q*_OSS_ as the C-related traps are more effective in reducing the 2DEG concentration. Again, reducing *t*_Chan_ leads to an almost constant *R*_ON_×*Q*_OSS_, as the decrease in *Q*_OSS_ is almost completely mirrored by an *R*_ON_ increase.

Figure 4b shows that barrier thickness (*t*_Bar_) increase only negligibly affects *R*_ON_ and *Q*_OSS_, while its reduction causes *R*_ON_ to slightly increase, therefore leading to a small *R*_ON_×*Q*_OSS_ increase of about 5% with a 50% reduction of *t*_Bar_. The fact that *R*_ON_ increases also prior to stress as shown in Figure 6b indicates that the increase due to *t*_Bar_ reduction is caused by an electrostatic effect (barrier thickness reduction causes a decrease in 2DEG concentration, due to a larger difference between the conduction band and the Fermi level at a given bias [1]).

Interestingly, Figure 4c shows that both *R*_ON_ and *Q*_OSS_ vary significantly with buffer thickness (*t*_Buf_). When *t*_Buf_ decreases, *R*_ON_ is reduced because for a given trap concentration the number of traps that become negatively charged is lower (because the volume is lower); therefore, the modulation of 2DEG by traps in the buffer is weaker and *R*_ON_ reduces. Opposite considerations apply when *t*_Buf_ increases. Regarding *Q*_OSS_, on top of the previous considerations about the variation of buffer traps, *t*_Buf_ reduction also increases *C*_OSS_; therefore, an additional increase of *Q*_OSS_ is observed; *t*_Buf_ increase instead reduces *Q*_OSS_ by about 20%. The effect of *t*_Buf_ on *Q*_OSS_ due to *C*_OSS_ modulation can be inferred from Figure 6c, which again shows results prior to stress (and thus allows one to appreciate the electrostatic contribution to the parameter variation). Overall, decreasing *t*_Buf_ causes an *R*_ON_×*Q*_OSS_ reduction of less than 10%; on the other hand, *R*_ON_×*Q*_OSS_ increases with increasing *t*_Buf_ up to almost 50% for a 100% *t*_Buf_ increase. Therefore, while reducing *t*_Buf_ might be beneficial to reduce *R*_ON_, increasing it leads to an overall penalty in device performance, because *R*_ON_ increase is more severe than *Q*_OSS_ reduction.

Figure 4d–f show the sensitivity on the length of the three field plates present in the structure (see Figure 2). Figure 4d shows that field plate 1 length (*L*_FP,1_) variations cause negligible variation of both *R*_ON_ and *Q*_OSS_. The slight *R*_ON_ reduction (*Q*_OSS_ increase) with increasing *L*_FP,1_ is attributed to the fact that a longer field plate increases the shielding effect of the ground bias (applied on the gate terminal in the OFF-state) against electric field peaks, thereby reducing the effect of trapped charge in the regions below the field plate itself. However, these variations are within 3% of the reference value, so little gain is to be expected from increasing *L*_FP,1_. Instead, when considering variations of field plate 2 length (*L*_FP,2_) as shown in Figure 4e, we can observe a more complex picture. Within ±50% *L*_FP,2_ variations, *R*_ON_ and *Q*_OSS_ vary symmetrically and by almost the same amount, as indicated by the fact that *R*_ON_×*Q*_OSS_ remains almost constant. However, increasing *L*_FP,2_ by 100% causes *R*_ON_ and *Q*_OSS_ to vary more considerably. While *R*_ON_ reduction is explained by the shielding effect discussed before for *L*_FP,1_, the considerable *Q*_OSS_ increase is mainly due to the increased coupling between the source and drain terminals, which increases *C*_DS_ (recall that this field plate is connected to the source terminal, see Figure 2). We conclude therefore that the effect of capacitive coupling between source and drain is more relevant to *Q*_OSS_ variation than the electric field shielding effect, as confirmed by the fact that *R*_ON_×*Q*_OSS_ increases. Again, the electrostatic increase of *C*_DS_ inducing *Q*_OSS_ increase can be appreciated from the results prior to stress, see Figure 6e. The effect of *L*_FP,3_ variations leads to similar results to those of the other field plates, as shown in Figure 4f. In this case, we also observe how *R*_ON_×*Q*_OSS_ remains almost constant (*L*_FP,3_ was not increased by more than 50% because further increase would cause an overlap of field plate 3 with the drain overhang shown in Figure 2).

As a general remark, we observe how field plates should be designed keeping in mind two constraints: on the one hand, field plates provide the beneficial effect of shielding from electric field peaks in the off-state; on the other hand, they increase the gate–drain and source–drain coupling (i.e., *C*_GD_ and *C*_DS_), which affect dynamic performance. Therefore, design of field plates is constrained by this trade-off, and depending on the target application either reducing or increasing field plate length might be more beneficial. Still, these considerations mostly apply to field plates 2 and 3, since field plate 1 is significantly shorter than the other ones, and much closer to the active region, therefore acting more as an electric field shield than anything else.

Concerning the modulation of the passivation thicknesses (*t*_Pas,1_–*t*_Pas,3_), Figure 4g–i show that both *R*_ON_ and *Q*_OSS_ variations are limited. Therefore, no significant advantage can be obtained from the variation of passivation thickness (provided that leakage is kept under control).

**Figure 5:**
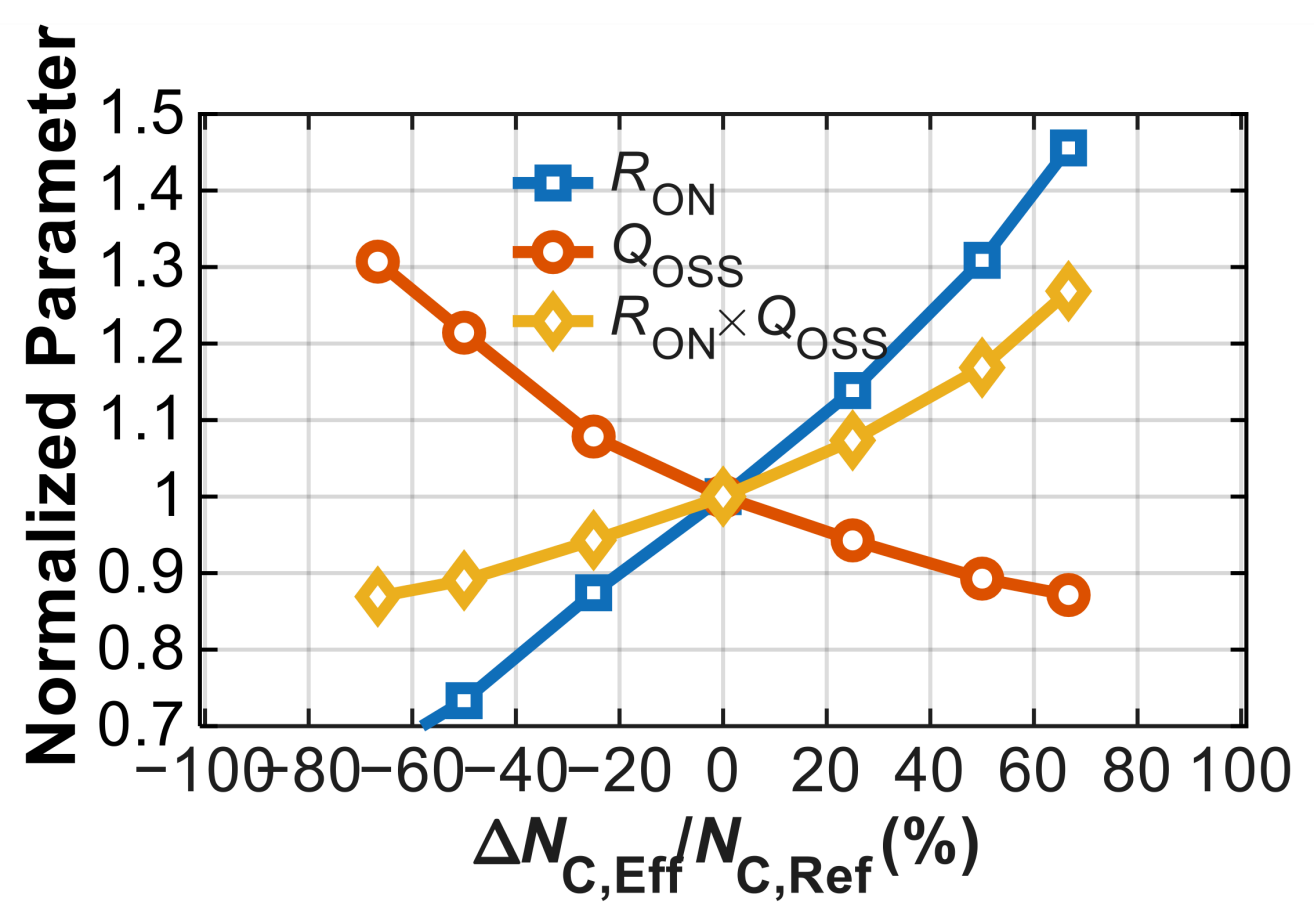
Sensitivity of *R*_ON_, *Q*_OSS_, and *R*_ON_*×Q*_OSS_ on variation of effective C-doping-related traps in the buffer (*N*_C,Eff_). *V*_D,STR_ = 400 V at *T* = 30 °C. Parameters were evaluated at the end of the stress phase.

Another important parameter that causes significant variation on *R*_ON_ and *Q*_OSS_ is the concentration of C-related traps in the buffer (*N*_C,Eff_). Variation of *N*_C,Eff_ is shown in Figure 5. An increase (decrease) in effective trap concentration increases (decreases) *R*_ON_ and decreases (increases) *Q*_OSS_; these results are expected since C-related traps affect both *R*_ON_ and *Q*_OSS_ as established in [8,9]. We notice how *R*_ON_×*Q*_OSS_ follows *N*_C,Eff_ variations; therefore, it would be advisable to reduce *N*_C,Eff_ as much as possible, provided that requirements on leakage and breakdown voltage are satisfied. We would like to point out that the picture above holds true as long as the increase in effective acceptor trap concentration occurs with increasing C-doping concentration. This might not hold true for very high C-doping concentration (i.e., higher than 10^19^ cm^−3^) for which the compensation by donors might also increase, overall leading to a reduced effective acceptor trap concentration [16].

Results in Figure 4 and Figure 5 were obtained at *T* = 30 °C and for a single *V*_DS,STR_ = 400 V. Temperature is known to accelerate dynamic *R*_ON_ and *Q*_OSS_ degradation, in the sense that at a given time, variation of both parameters is larger in magnitude compared to the *T* = 30 °C case. However, after 1000 s stress, as can be appreciated from Figure 2, dynamic *R*_ON_ and *Q*_OSS_ have reached a steady-state condition; therefore, from a qualitative point of view the trends in Figure 4 and Figure 5 are expected to remain unaffected by temperature. Regarding *V*_DS,STR_ variation, the picture is more complex as one has to consider that both dynamic *R*_ON_ and *Q*_OSS_ vary non-monotonically with *V*_DS,STR_, as reported in [8,9]. Therefore, the choice of *V*_DS,STR_ will change the magnitude of degradation of both parameters, but not the qualitative behavior.

Results in Figure 4 and Figure 5 are obtained with the substrate grounded. The expected impact of having a floating substrate is to exacerbate the magnitude of dynamic *R*_ON_ (and consequently *Q*_OSS_), as suggested in [17]. Having the substrate grounded, the device configuration in this work resembles that of a low-side device in a half-bridge configuration. While not specifically addressed in this work, in the case of monolithic integration of a half-bridge on a single substrate, the high-side device is expected to experience worse degradation in magnitude compared to the low-side one [18,19].

**Figure 6:**
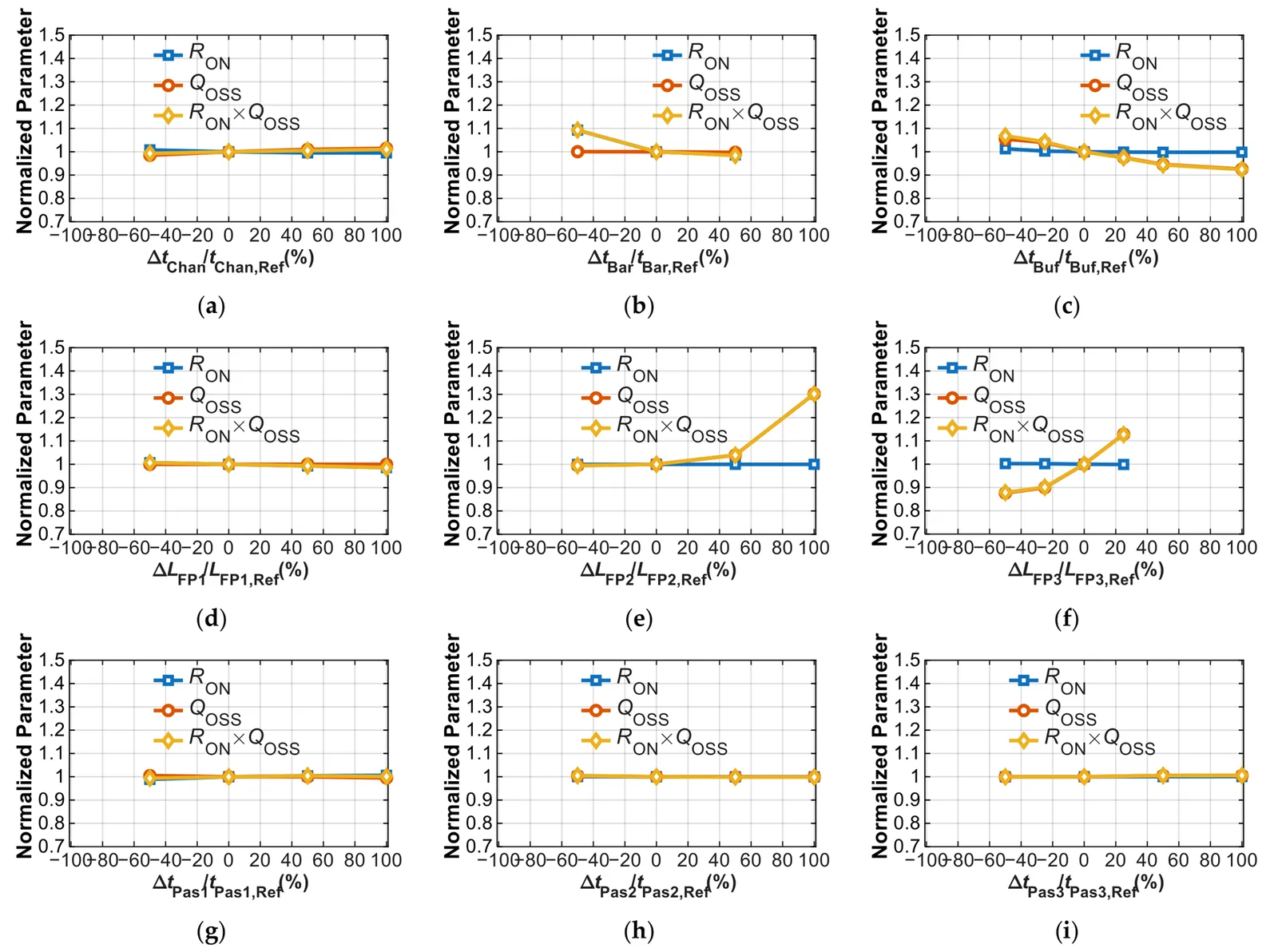
Sensitivity of *R*_ON_, *Q*_OSS_, and *R*_ON_×*Q*_OSS_ on variation of geometric parameters: (**a**) channel thickness, *t*_Chan_; (**b**) barrier thickness, *t*_Bar_; (**c**) buffer thickness, *t*_Buf_; (**d**) field plate 1 length (*L*_FP,1_); (**e**) field plate 2 length (*L*_FP,2_); (**f**) field plate 3 length (*L*_FP,3_); (**g**) passivation 1 thickness, *t*_Pas,1_; (**h**) passivation 2 thickness, *t*_Pas,2_; (**i**) passivation 3 thickness, *t*_Pas,3_. Parameters were evaluated prior to the stress phase at *T* = 30 °C.

### 3.2. Results Prior to Stress

Figure 6 reports the simulation results of the sensitivity analysis on *R*_ON_ and *Q*_OSS_ obtained prior to the stress test (i.e., as specified in Section 2, these results are obtained after applying stress for 10 μs, which is short enough not to induce any measurable effect of dynamic *R*_ON_ and *Q*_OSS_ at *T* = 30 °C). This data complements the one shown in Figure 4, and the comparison allows assessing the effect of prolonged operation on *R*_ON_, *Q*_OSS_, and on the *R*_ON_×*Q*_OSS_ FOM. Generally, we observe from Figure 6 how *R*_ON_ and *Q*_OSS_ are much less affected by parameter variations compared to Figure 4. This is attributed to the fact that the effect of C-related traps manifests itself only with the passing of time; therefore, *R*_ON_ and *Q*_OSS_ variations prior to stress (for instance, with *t*_Bar_ variation) have only to do with the electrostatics of the device, and not with C-related trapping effects. However, in some cases *Q*_OSS_ variation is significant even prior to stress due to electrostatics, see for instance Figure 6c,e,f. These variations have to do with device capacitance modulation induced by geometric parameter variations.

**Figure 7:**
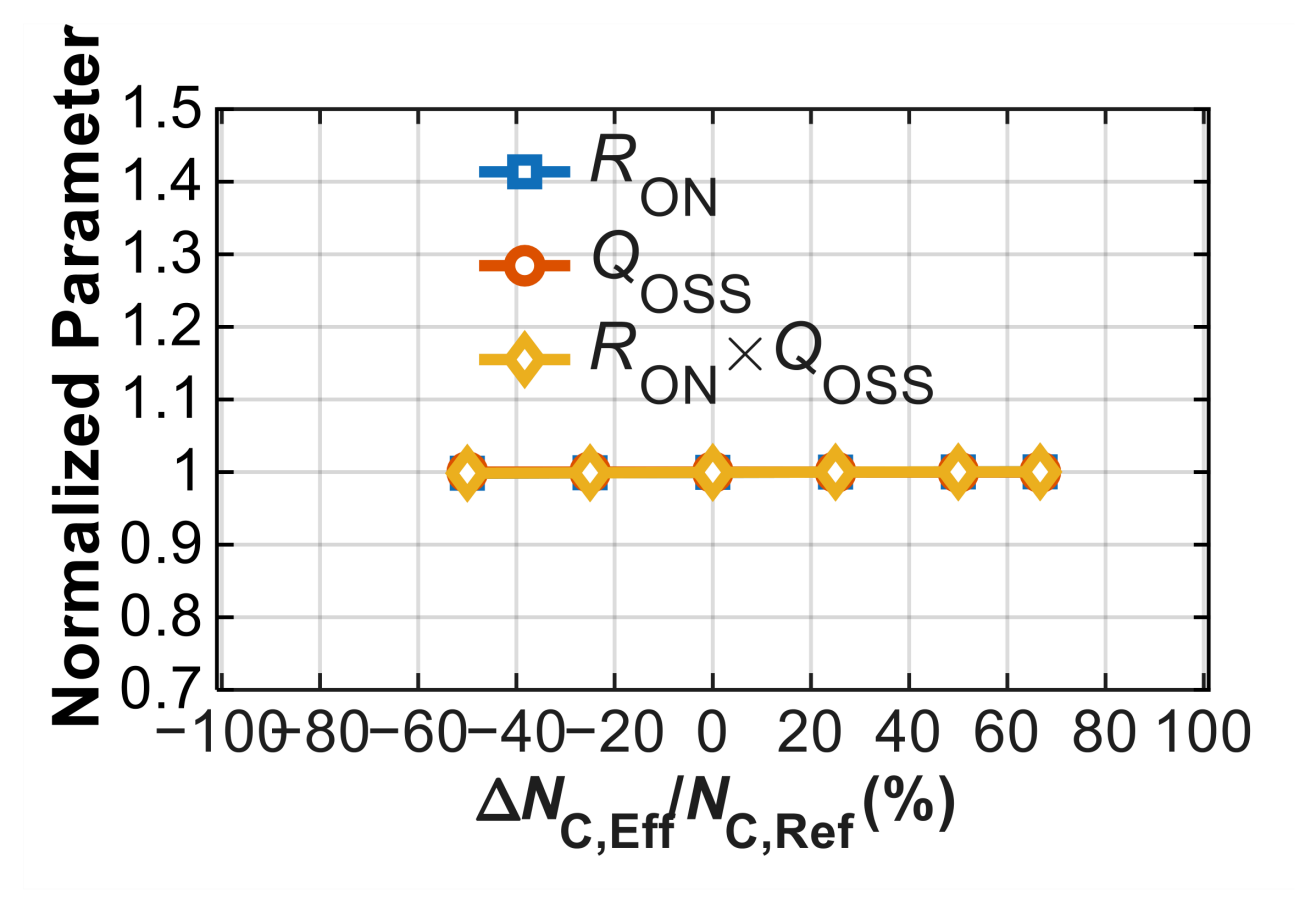
Sensitivity of *R*_ON_, *Q*_OSS_, and *R*_ON_×*Q*_OSS_ on variation of effective C-doping-related traps in the buffer (*N*_C,Eff_). Parameters were evaluated prior to the stress phase at *T* = 30 °C.

Lastly, Figure 7 shows the influence on the parameters of *N*_C,Eff_ variation prior to the stress phase. Interestingly, both *R*_ON_ and *Q*_OSS_ remain almost insensitive to variations because the trap time constant is longer than the evaluation time window (i.e., carbon has little effect on *R*_ON_ and *Q*_OSS_ prior to stress). In practice, data presented in Figure 4 and Figure 5 are more important to the designer as the device will inevitably incur degradation during operation.

### 3.3. Discussion

From the results obtained so far, we observe that reducing *R*_ON_×*Q*_OSS_ is possible by increasing channel thickness, decreasing buffer thickness, reducing source-connected field plate length, or reducing effective carbon doping concentration. Table 3 summarizes the variations obtained in terms of *R*_ON_, *Q*_OSS_, and *R*_ON_×*Q*_OSS_ when performing the above variations. A comparison of the values obtained after and prior to stress allows one to immediately quantify how much of the variation is due to trapping effects or to electrostatic effects. In Table 3, we add a comparison of the breakdown voltage (*V*_BR_) and leakage current (*I*_D,Leak_) variation obtained with respect to the nominal case for each of the main parameters of interest identified with the previous analysis. *V*_BR_ was defined as the *V*_DS_ at which *I*_D_ = 1 mA/mm when *V*_GS_ = 0 V (for the nominal case, *V*_BR_ = 877 V), whereas *I*_D,Leak_ was taken at *V*_DS_ = 650 V (for the nominal case, *I*_D,Leak_ = 1.33 nA/mm). From Table 3 we can observe how increasing *t*_Chan_ or *L*_FP,2_ length gives negligible variation in *V*_BR_, and reduction of *N*_C,Eff_ leads to about 5% reduction in *V*_BR_. Only when reducing *t*_Buf_ by 50% does *V*_BR_ reduce significantly by 39%. Concerning *I*_D,Leak_ instead, increasing *t*_Chan_ leads to about 1.44 nA/mm leakage current, whereas reducing *L*_FP,2_ almost leaves *I*_D,Leak_ unaffected. When decreasing *t*_Buf_ by 50%, *I*_D,Leak_ is not defined as the device breaks down below 650 V; for reduced *N*_C,Eff_ instead *I*_D,Leak_ increases by about 26 times to 35.9 nA/mm (this increase is in line with the difference between typical and maximum leakage current of commercial devices listed in Table 2). These indications give a clear picture to a designer of the trade-off between *R*_ON_×*Q*_OSS_, *V*_BR_, and *I*_D,Leak_ upon critical parameter variations.

## 4. Conclusions

We analyzed by means of numerical simulations the device design implications on the *R*_ON_×*Q*_OSS_ Figure of Merit (FOM) in p-GaN power HEMTs to be employed in soft-switching converters. *R*_ON_ and *Q*_OSS_ are strongly affected by C-related traps in the buffer; hence, device design can significantly affect their values by varying the amount of modulated negative trapped charge in the buffer. The analysis reveals that reducing *R*_ON_×*Q*_OSS_ is possible by either: increasing channel thickness, decreasing buffer thickness, reducing source-connected field plate length, or reducing effective carbon doping concentration. These indications stem from the investigated device structure and parameter ranges, therefore they should be intended as simulation trends rather than general optimization rules.

## Figures and Tables

**Table 3:** Summary of *R*_ON_, *Q*_OSS_, *R*_ON_×*Q*_OSS_, and *V*_BR_ variation upon variation of the most critical design parameters obtained from Figure 4 and Figure 5 (values between brackets are those obtained from Figure 6 and Figure 7).

Parameter Variation	*R*_ON_ Variation	*Q*_OSS_ Variation	*R*_ON_×*Q*_OSS_ Variation	*V*_BR_ Variation	*I*_D,Leak_ Variation
+100% *t*_Chan_	−11% (<1%)	+8.6% (+1.5%)	−3% (+1%)	<1%	+8.3%
−50% *t*_Buf_	−30% (+1.3%)	+35% (+5.4%)	−5% (+6.8%)	−39%	N.A. *
−50% *L*_FP,2_	+3.5% (<1%)	−5% (<1%)	−2% (<1%)	<1%	<1%
−66.7% *N*_C,Eff_	−33.5% (<1%)	+30.7% (<1%)	−13% (<1%)	−5%	×26 **

* *I*_D,Leak_ is not defined because the device breakdown voltage is less than 650 V in this case. ** This increase is in line with the difference between typical and maximum leakage current of commercial devices listed in Table 2.

## Data Availability

The original contributions presented in this study are included in the article. Further inquiries can be directed to the corresponding author.
